# Bacteriophages as Potential Anti-Pathogenic Agents for Intestinal Health of Weaned Piglets in the Post-Antibiotic Era: An Updated Review

**DOI:** 10.3390/ani15121713

**Published:** 2025-06-10

**Authors:** Jun Chen, Jiajun Han, Zheng Yang, Wenyue Zhou, Yuyong He, Xingping Chen, Xin Li, Tiande Zou, Jinming You

**Affiliations:** Jiangxi Province Key Laboratory of Animal Nutrition and Feed, Jiangxi Province Key Innovation Center of Integration in Production and Education for High-Quality and Safe Livestock and Poultry, Jiangxi Agricultural University, Nanchang 330045, China; junchen@jxau.edu.cn (J.C.); 19845198016@163.com (J.H.); 15737566134@163.com (Z.Y.); 18348392842@163.com (W.Z.); wlkh1012@163.com (Y.H.); cxp0315@jxau.edu.cn (X.C.); lixin@jxau.edu.cn (X.L.); tiandezou@jxau.edu.cn (T.Z.)

**Keywords:** antibiotic resistance, anti-pathogenic agents, bacteriophages, diarrhea, growth performance, intestinal health, post-antibiotic era, swine industry, weaned piglets

## Abstract

Background: The use of in-feed antibiotics was banned in numerous countries within the animal production industry as a result of the emergence of antibiotic-resistant bacteria and the presence of residual antibiotics. Bacteriophages, which are viruses that infect host bacterial cells, are considered the natural predators of bacteria. Over the past two decades, bacteriophages have garnered increasing attention for their potential in controlling pathogenic bacteria in weaned piglets. Aims: The aim of this overview was to update the progress of bacteriophage application in weaned piglets. Methods: For the section on bacteriophage application in weaned piglets, a systematic search was performed to identify relevant articles published before June 2025 in databases such as Web of Science. Results: In this review, we provide a brief overview of bacteriophages, followed by a summary of the isolation of specific bacteriophages in weaned piglets. In addition, we have summarized the application progress of bacteriophages in weaned piglets, including the effects of oral administration or dietary supplementation with bacteriophages on growth performance, diarrhea characteristics, intestinal morphology, intestinal pH, nutrient digestibility, inflammatory response, intestinal barrier function, and intestinal microecology. Conclusions: This updated overview novelly highlights the potential of bacteriophages as anti-pathogenic agents in mitigating infections caused by pathogenic bacteria in weaned piglets. This review could provide a scientific basis for controlling pathogenic bacteria infections in weaned piglets in the post-antibiotic era.

## Simple Summary

Due to their immature immune and gastrointestinal systems, weaned piglets are highly susceptible to intestinal pathogens. The ever-increasing emergence of antibiotic-resistant bacterial strains has prompted swine researchers to renew their focus on bacteriophages as potential antimicrobial agents. This review systematically updated the application of bacteriophages in weaned piglets, which could provide practical guidance for controlling diarrhea and intestinal health problems in weaned piglets.

## 1. Introduction

In-feed antibiotics have long been utilized in the swine industry to enhance growth performance, optimize feed efficiency, and prevent diseases [1]. However, the use of in-feed antibiotics was banned in numerous countries [2]. In 1986, Sweden completely banned the use of growth-promoting antibiotics in livestock and poultry feed, becoming the first country to prohibit the use of antibiotics as growth promoters [3]. Thereafter, the European Union (EU) revoked its authorization for the use of antibiotics as growth promoters for animals, with the ban taking effect on 1 January 2006 [4]. China, the world’s largest producer of pigs and consumer of pork, discontinued all growth-promoting pharmaceutical feed additives in animal production, with the exception of traditional Chinese medicines, effective 1 January 2020 [5]. One of the key reasons for prohibiting in-feed antibiotics is the emergence of antibiotic resistance [6]. For instance, Holmer et al. (2019) reported that nearly 70% of *Escherichia coli* (*E. coli*) strains isolated from pigs in Denmark exhibited resistance to tetracycline and streptomycin, alongside a significant increase in florfenicol resistance [7]. Another crucial reason for banning in-feed antibiotics is the presence of residual antibiotics in animal tissues, which poses risks to both animal welfare [8] and food safety [9]. These residues, particularly in organs such as the liver and kidneys, which are primary sites of drug metabolism, can impair normal physiological functions [10].

Diarrhea and intestinal health problems in weaned piglets pose a major challenge to the swine industry [11,12]. Pathogenic bacterial infection is a significant cause of intestinal diseases and diarrhea in weaned piglets, particularly *E. coli* and *Salmonella typhimurium* [13,14]. In the post-antibiotics era, the management of diarrhea and intestinal health in weaned piglets presents a significant challenge in the global pig industry [15]. It is imperative to develop alternative products for the treatment of pathogenic bacterial infections in weaned piglets [16]. The antimicrobial properties of bacteriophages have been employed to address bacterial infections since their identification in the early twentieth century; however, their therapeutic application was later overshadowed by the widespread use of antibiotics [17]. The emergence of antibiotic resistance, especially the widespread dissemination of multidrug-resistant strains, has prompted a renewed global interest in utilizing bacteriophages for the treatment of bacterial infections in humans and animals [18,19].

Therefore, the aim of this review was to update recent advancements in bacteriophage applications in weaned piglets. In this review, we first provide a brief overview of bacteriophages and then summarize the isolation of specific bacteriophages targeting enteric pathogens in weaned piglets. Finally, we review the application progress of bacteriophages in weaned piglets, focusing on their effects on growth performance, diarrhea characteristics, intestinal morphology, intestinal pH, nutrient digestibility, inflammatory response, intestinal barrier function, and intestinal microecology. For the section on bacteriophage application in weaned piglets, a systematic search was performed to identify relevant articles published before June 2025 in the Web of Science, PubMed, and Google Scholar databases. The search terms included “bacteriophage + weaned piglets”, “phage + weaned piglets”, “bacteriophage + nursery piglets”, and “phage + nursery piglets”. Studies focusing on other swine phases were excluded, with only research involving weaned or nursery piglets included to ensure relevance to bacteriophage application in this specific group.

## 2. An Overview of Bacteriophages

Bacteriophages are extremely abundant in bacteria-rich environments [20], with an estimated population of 10^31^ bacteriophages present in the biosphere, a number that is 10 times greater than the total bacterial population [21].

Bacteriophages can be categorized as either virulent (lytic) or temperate based on their replication types [20,22]. Upon infecting the bacterial cell, virulent bacteriophages manipulate the cellular machinery, degrade the host bacterial DNA, and synthesize bacteriophage DNA and proteins to facilitate rapid replication [18]. This process culminates in cell lysis, releasing hundreds of progeny bacteriophages that subsequently infect additional host bacteria [18]. In contrast, after infecting the host bacteria, temperate bacteriophage DNA integrates into the bacterial chromosome, forming a probacteriophage instead of generating new viral particles [23]. The resulting probacteriophage synchronously replicates with each host cell division. Subsequently, an external stimulus, such as ionizing radiation or a specific chemical agent, induces the probacteriophage to initiate the lytic cycle [23]. Temperate bacteriophages are generally avoided in anti-pathogenic applications due to their capacity to facilitate gene transfer through specialized transduction, which may promote the spread of antibiotic resistance or increase bacterial pathogenicity [18,23]. Furthermore, upon integration into the bacterial genome, they may demonstrate superinfection immunity, thereby rendering subsequent bacteriophage treatments ineffective [18]. By contrast, virulent bacteriophages are preferred as efficient antibacterial agents due to their rapid lethality toward target host bacterial cells [23].

## 3. Isolation of Specific Bacteriophages Targeting Enteric Pathogenic Bacteria in Weaned Piglets

For the effective isolation of bacteriophages, the selection of appropriate host bacterial strains and the source of collected samples are two crucial factors [24]. In weaned piglets, bacterial infections caused by *E. coli* [16] and *Salmonella typhimurium* [16,25] are of significant concern. Given the highly specific nature of bacteriophages targeting particular bacterial species, the primary pathogenic strains, namely *E. coli* and *Salmonella typhimurium*, are chosen as host bacteria for the isolation process from the collected samples [16]. Although bacteriophages are ubiquitous in the environment, the most effective bacteriophages are typically isolated from or near the site of infection [26,27]. Therefore, samples for the isolation of bacteriophages targeting intestinal pathogens in weaned piglets were predominantly collected from sewage, manure, and feces in pig farms, as well as from food processing plants, drained water from meat stalls, and dissected areas in local markets. Table 1 summarizes the isolation of specific bacteriophages targeting intestinal pathogenic bacteria in weaned piglets.

### 3.1. Isolation of Bacteriophage Targeting E. coli

In the field of bacteriophage isolation targeting *E. coli*, various studies have demonstrated the successful isolation of bacteriophages from or near the site of infection. For instance, Mao et al. (2023) used *E. coli* GXXW-1103 as the host bacterial strain and successfully isolated a bacteriophage from pig farm sewage [28]. Similarly, Imklin et al. (2022) utilized 14 *E. coli* strains (M158, M170, M171, M179, M181, M184, M187, M209, M226, M240, M241, M242, M243, and M245) as the host bacterial strain to isolate six bacteriophages (vB_EcoM-RPN170, vB_EcoM-RPN171, vB_EcoM-RPN187, vB_EcoM-RPN226, vB_EcoM-RPN242, and vB_EcoP-RPN243) from pig manure, drained water from meat stalls, and dissected areas in local markets [29]. In earlier research, Lee et al. (2017) used ETEC K88 as the host bacterial strain and isolated bacteriophage L86 from pig manure collected from a commercial farm [30]. Likewise, Han et al. (2016) utilized ETEC K88 and K99 as host bacterial strains to isolate specific bacteriophages from fecal samples of grower pigs aged 30 to 70 days on a commercial swine farm [31]. Additionally, Lin et al. (2021) employed 87 *E. coli* strains as host bacteria and isolated an effective bacteriophage, bacteriophage C1, from pig farm fecal samples [12]. This bacteriophage was subsequently reported for its application in weaned piglets [32]. Furthermore, Jamalludeen et al. (2007) isolated the lytic bacteriophage CJ12 using ETEC JG280 as the host bacterial strain from pig farm sewage [33], and its application in weaned piglets was later documented [34]. In a more recent study, Zhou et al. (2022) used *E. coli* O157:H7 GN07 as the host bacterium strain to isolate bacteriophage EP01 from pig farm sewage [35], with a subsequent study reporting its potential application in weaned piglets [16]. Collectively, these studies highlight the diverse range of *E. coli*-specific bacteriophages that have been successfully isolated from various pig-related environments, underscoring their potential as anti-*E. coli* agents in weaned piglets [12,16,28,29,30,31,32,33,34,35].

### 3.2. Isolation of Bacteriophage Targeting Salmonella typhimurium

In the pursuit of isolating *Salmonella typhimurium*-specific bacteriophages, several studies have employed various host bacterial strains and sample sources to achieve successful isolation. For example, Li et al. (2024) utilized *Salmonella typhimurium* SM022 as the host bacterial strain and successfully isolated the *Salmonella* bacteriophage NJ12 from pig farm sewage [16]. Similarly, Thanki et al. (2019) utilized 12 *Salmonella* strains as host bacteria and isolated two bacteriophages, SPFM10 and SPFM14, from a food processing plant and a pig farm [36]. These bacteriophages were later reported for their application in weaned piglets [38]. In another study, Won et al. (2021) used *Salmonella typhimurium* as the host bacterial strain and isolated a bacteriophage *(Salmonella typhimurium*-specific bacteriophage STP-1) from pig manure obtained from a sewage treatment plant at a commercial swine farm [39]. Additionally, using six *Salmonella* strains as host bacteria, Seo et al. (2018) isolated 16 lytic bacteriophages (bacteriophage SEP-1, SGP-1, STP-1, SS3eP-1, EK99P-1, SalTP-2, SChP-1, SAP-1, SAP-2, E41P-1, EK88P-1, CPP-3, and CPP-5) from sewage and fecal samples collected from five pig farms [40]. Therefore, these studies demonstrate the feasibility and diversity of isolating *Salmonella typhimurium*-specific bacteriophages from various pig-related environmental sources, highlighting their potential for application in veterinary medicine, particularly in the context of weaned piglets.

## 4. The Application of Bacteriophages in Weaned Piglets

In recent years, a growing body of research has been conducted to investigate the potential applications of bacteriophages in weaned piglets. A comprehensive summary of the applications of bacteriophages in weaned piglets is provided in Table 2.

### 4.1. Effects on Growth Performance of Weaned Piglets

Bacteriophages have been shown to enhance the growth performance of weaned piglets in models challenged with *E. coli* and/or *Salmonella typhimurium*. Mao et al. (2023) demonstrated that the oral administration of a microencapsulated bacteriophage at a concentration of 1 × 10^9^ PFU/mL, delivered in 5 mL doses daily for 7 days, significantly increased the average daily gain of piglets challenged with *E. coli* GXXW-1103 [28]. In addition to oral administration, dietary supplementation has emerged as another effective approach for delivering bacteriophages to weaned piglets. For instance, Lee et al. (2017) reported that weaned piglets fed a diet supplemented with 1 × 10^7^ PFU/kg of bacteriophages for 14 days exhibited significant increases in both final body weight and average daily gain under ETEC K88 challenge [30]. Also, under the co-challenge of ETEC K88 and K99, dietary supplementation with ETEC K88-specific and K99-specific bacteriophages at a concentration of 1 × 10^9^ PFU of each bacteriophage per kilogram of feed for 7 days significantly elevated final body weight and average daily gain in weaned piglets [31]. Additionally, Won et al. (2021) found that dietary supplementation with 1 × 10^9^ PFU/kg of *Salmonella typhimurium*-specific bacteriophage for 14 days resulted in increased final body weight and average daily gain in weaned piglets challenged with *Salmonella typhimurium* CTC1110 [39]. These findings highlight the versatility and efficacy of bacteriophage administration through both oral and dietary routes in improving growth performance in weaned piglets under *E. coli* and/or *Salmonella typhimurium* challenges.

Bacteriophages have also been reported to improve the growth performance of weaned piglets raised in non-sanitary environments or under practical production conditions. In a non-sanitary environment, weaned piglets fed a diet supplemented with a bacteriophage cocktail (1 × 10^8^ PFU/kg) for 14 days exhibited significant increases in average daily gain and gain-to-feed ratio [41]. Consistent with these findings, Hosseindoust et al. (2017) reported that dietary supplementation with a bacteriophage cocktail (1 × 10^9^ PFU of each bacteriophage per gram, administered at a dosage of 1 g/kg) for 35 days significantly increased average daily gain and gain-to-feed ratio in weaned piglets raised in a contaminated environment [42]. Under practical production conditions, our previous study showed that dietary supplementation with a bacteriophage cocktail (1 × 10^6^ PFU/g, at a dosage of 0.4 g/kg) significantly increased final body weight, average daily feed intake, average daily gain, and gain-to-feed ratio in weaned piglets [44]. Similarly, Hosseindoust et al. (2017) and Kim et al. (2017) reported that feeding weaned piglets diets supplemented with bacteriophage cocktails (at concentrations of 1 × 10^9^ PFU/g and dosages of 1 g/kg and 1.5 g/kg, respectively) enhanced their average daily gain [45,46]. Consistently, Lee et al. (2016) also observed increased average daily gain and gain-to-feed ratio in weaned piglets fed a diet supplemented with a bacteriophage cocktail (1 × 10^9^ PFU/g, at a dosage of 1 g/kg) [47]. These studies suggest the potential of bacteriophages in enhancing growth performance in weaned piglets under non-sanitary environments or practical production conditions, where these piglets may also face challenges from pathogenic bacteria.

However, it should be noted that most of these experiments were limited to laboratory-scale studies, with only a small number conducted in practical pig farm settings involving small groups of pigs. Under laboratory conditions, piglet models infected with gut pathogens like *E. coli* and *Salmonella typhimurium* exhibit limitations. In practice, piglets may or may not encounter these pathogens across different farms and can also experience co-infections with multiple bacteria, adding complexity to the situation. Therefore, further research is warranted to evaluate the efficacy of bacteriophages in diverse practical pig farm settings, including large groups of pigs under varying conditions such as severe diarrhea, differing sanitary environments, and seasonal variations.

### 4.2. Effects on Diarrhea Characteristics of Weaned Piglets

Several studies have demonstrated the efficacy of bacteriophages in mitigating diarrhea phenotypes in weaned piglets. For instance, Cha et al. (2012) found that dietary supplementation with ETEC-specific bacteriophage reduced the percentage of diarrhea in weaned piglets challenged with ETEC JG280 [34]. Similarly, Han et al. (2016) reported that dietary supplementation with bacteriophages specific to ETEC K88 and ETEC K99 reduced rectal temperature and fecal consistency scores in weaned piglets co-challenged with these pathogens (ETEC K88 and ETEC K99) [31]. More recently, Li et al. (2024) observed that oral administration of a microencapsulated bacteriophage cocktail decreased diarrhea incidence in weaned piglets co-challenged with *E. coli* O157:H7 and *Salmonella typhimurium* SM022 [16].

In a non-sanitary environment, Choi et al. (2023) noted that dietary supplementation with a bacteriophage cocktail reduced the fecal score in weaned piglets [41]. Consistently, Hosseindoust et al. (2017) demonstrated that dietary supplementation with a bacteriophage cocktail reduced the fecal score in weaned piglets raised in a contaminated environment [42]. Kingkan et al. (2023) further showed that dietary supplementation with a bacteriophage cocktail resulted in lower fecal scores compared to weaned piglets fed antibiotics [43]. In our previous study, we also found that incorporating a bacteriophage cocktail into the diet decreased diarrhea incidence in weaned piglets [44]. Additional studies have also highlighted the benefits of bacteriophage supplementation. Hosseindoust et al. (2017) documented a reduction in fecal scores of weaned piglets following dietary supplementation with a bacteriophage cocktail [45]. Similarly, Kim et al. (2017) reported that a bacteriophage cocktail reduced the fecal score in weaned piglets [46]. Lee et al. (2016) demonstrated that incorporating a bacteriophage cocktail into the diet reduced the fecal score in weaned piglets, regardless of the presence or absence of 0.34% zinc oxide [47]. Collectively, these studies underscore the potential of bacteriophages in reducing diarrhea incidence and improving fecal consistency in weaned piglets, particularly under challenging conditions such as pathogen exposure and non-sanitary environments.

### 4.3. Effects on Intestinal Morphology of Weaned Piglets

Several studies have investigated the effects of bacteriophages on intestinal morphology in weaned piglets. For instance, Lee et al. (2017) found that dietary supplementation with ETEC K88-selective bacteriophage increased ileal villus height, villus height to crypt depth ratio, goblet cell density in ileal villi and crypts, and decreased ileal crypt depth in weaned piglets challenged with ETEC K88 [30]. Similarly, Won et al. (2021) reported that dietary supplementation with *Salmonella typhimurium*-specific bacteriophage for 14 days increased jejunal villous height, villus height to crypt depth ratio, and goblet cell density of villi in weaned piglets challenged with *Salmonella typhimurium* CTC1110 [39]. Under a non-sanitary rearing condition, Choi et al. (2023) observed that dietary supplementation with a bacteriophage cocktail for 14 days increased villus height in the duodenum of weaned piglets [41]. Hosseindoust et al. (2017) also found that administering a bacteriophage cocktail as a dietary supplement for 35 days increased jejunal villus height in weaned piglets exposed to a contaminated environment [42].

Additionally, our previous study demonstrated that dietary supplementation with a bacteriophage cocktail for 21 days increased villus height (in the jejunum and ileum) and villus height to crypt depth ratio (in the duodenum, jejunum, and ileum), while decreasing crypt depth (in the jejunum and ileum) in weaned piglets [44]. Additional studies have further supported these findings. Hosseindoust et al. (2017) reported that feeding weaned piglets a bacteriophage cocktail for 35 days increased villus height in the duodenum and jejunum [45]. Similarly, Kim et al. (2017) found that a 35-day diet supplemented with a bacteriophage cocktail increased villous height in both the duodenum and jejunum [46]. Lee et al. (2016) demonstrated that dietary supplementation with a bacteriophage cocktail, with or without 0.34% zinc oxide, for 35 days increased villus height in the duodenum and jejunum of weaned piglets [47]. However, when compared to the antibiotics group, dietary supplementation with a bacteriophage cocktail for 21 days resulted in a reduction in duodenal villus height-to-crypt depth ratio, jejunal villus height, crypt depth, and villus height-to-crypt depth ratio in weaned piglets [43]. Taken together, those results highlight the potential of bacteriophage supplementation in improving intestinal morphology in weaned piglets, particularly in enhancing villus height and villus height to crypt depth ratio, which are critical indicators of intestinal health and digestive function. However, the effects of bacteriophages may not yet be comparable to those of in-feed antibiotics in terms of intestinal morphology [43].

### 4.4. Effects on Intestinal pH and Nutrient Digestibility of Weaned Piglets

One key factor influencing gut health is the pH of the intestinal environment, which can affect microbial balance, nutrient absorption, and overall digestive function [48]. Modulating the gut environment through dietary interventions is a promising strategy to enhance gut health and resilience in weaned piglets. Lee et al. (2017) reported that weaned piglets fed a diet supplemented with a bacteriophage for 14 days exhibited a significant decrease in the pH of the digesta in the colon of piglets infected with ETEC K88 [30]. Consistent with these findings, Han et al. (2016) demonstrated that under a co-challenge with both ETEC K88 and K99, dietary supplementation with ETEC K88-specific and K99-specific bacteriophages for 7 days also resulted in a decreased pH of the digesta in the colon of weaned piglets [31]. These studies highlight the potential of bacteriophage supplementation to improve gut health in weaned piglets by modulating the intestinal pH.

Nutrient digestibility is a critical factor influencing the growth and health of weaned piglets [49]. Hosseindoust et al. (2017) discovered that dietary supplementation with a bacteriophage cocktail at a dosage of 1 g/kg (1 × 10^9^ PFU/g) for 35 days resulted in an increased apparent total tract digestibility of crude protein in weaned piglets raised in a contaminated environment [42]. Hosseindoust et al. (2017) also found that dietary supplementation with a bacteriophage cocktail at a dosage of 1 g/kg (1 ×10^9^ PFU/g) resulted in an increase in the apparent total tract digestibility of dry matter and crude protein in weaned piglets [45]. Consistently, Kim et al. (2017) found that administering a bacteriophage cocktail as a dietary supplement to weaned piglets at a dosage of 1.5 g/kg (1 × 10^9^ PFU/g) for a period of 35 days resulted in a significant improvement in the apparent total tract digestibility of both dry matter and crude protein [46]. Additionally, Lee et al. (2016) found that dietary supplementation with a bacteriophage cocktail for a period of 35 days resulted in a significant increase in the apparent total tract digestibility of dry matter and crude protein, regardless of whether or not it was supplemented with 0.34% zinc oxide [47]. These studies highlight the potential of bacteriophage supplementation to enhance nutrient digestibility in weaned piglets, particularly in challenging rearing conditions.

### 4.5. Effects on Intestinal Barrier Function of Weaned Piglets

Intestinal barrier function is critical for maintaining gut health and overall well-being in animals [50,51,52], particularly during the early post-weaning period when piglets are highly susceptible to gastrointestinal disturbances [53]. Recent research has explored the potential of bacteriophages to enhance these aspects of gut health. For instance, Liu et al. (2024) observed significant elevations in the levels of tight junction proteins, including ZO-1, Claudin-1, and MUC2, in the jejunum and colon of piglets administered a bacteriophage cocktail [32]. These findings suggest that bacteriophages may enhance intestinal barrier function by promoting the expression of key proteins involved in maintaining tight junction integrity. Furthermore, our previous study found that incorporating a bacteriophage cocktail into the diet led to reductions in serum D-lactate and diamine oxidase levels, both of which are indicators of gut barrier dysfunction and intestinal permeability [44]. This suggests that bacteriophage supplementation may help mitigate gut leakage. Our previous study also observed increased levels of intestinal trefoil factor, secretory immunoglobulin A (sIgA), and tumor growth factor-α (TGF-α) in piglets receiving a bacteriophage cocktail [44]. These molecules play crucial roles in maintaining intestinal homeostasis, mucosal repair, and immune regulation. Elevated levels of these factors indicate enhanced intestinal defense mechanisms and a more robust mucosal barrier. Moreover, we observed elevated jejunal mucosal mRNA levels of *Occludin* and *Claudin-1*, as well as increased protein expression levels of Occludin in weaned piglets supplemented with the bacteriophage cocktail [44]. The protective effect of bacteriophages on *E. coli*-induced intestinal barrier dysfunction was also demonstrated in a porcine intestinal epithelial cell line (IPEC-J2) model [54]. These results further support the notion that bacteriophage supplementation can modulate the expression of tight junction proteins at both the transcriptional and translational levels, thereby strengthening the intestinal barrier. Collectively, these findings highlight the multifaceted benefits of bacteriophage supplementation in improving intestinal barrier function and reducing markers of gut permeability in weaned piglets.

### 4.6. Effects on Inflammation Response of Weaned Piglets

In recent years, the potential of bacteriophages to modulate the immune response has gained significant attention as a novel and promising strategy in veterinary medicine and animal nutrition. Several studies have explored the effects of bacteriophages on the immune response in weaned piglets. Most recently, Li et al. (2024) demonstrated that oral administration of a microencapsulated bacteriophage cocktail decreased neutrophil levels in the blood of weaned piglets challenged with *E. coli* O157:H7 and *Salmonella typhimurium* SM022 [16]. Similarly, Lee et al. (2017) found that dietary supplementation with an ETEC K88-selective bacteriophage reduced serum TNF-α levels in weaned piglets challenged with ETEC K88 [30]. TNF-α is a pro-inflammatory cytokine that plays a central role in the inflammatory cascade, and its reduction indicates a potential anti-inflammatory effect of bacteriophage supplementation. Choi et al. (2023) further expanded the understanding of bacteriophage effects by demonstrating that dietary supplementation with a bacteriophage cocktail decreased serum levels of IL-1β, IL-6, TNF-α, and Zonulin, as well as jejunal myeloperoxidase activity in weaned piglets raised in a non-sanitary environment [41]. These results highlight the potential of bacteriophages to modulate both systemic and local intestinal inflammation, which is particularly relevant in challenging rearing conditions.

Consistent with these observations, our previous study also noted reduced levels of IL-1β and TNF-α, along with increased IL-10 levels in the serum of piglets fed a diet supplemented with bacteriophages [44]. Additionally, we observed heightened mRNA levels of jejunal mucosa toll-like receptors *TLR-2*, *TLR-4*, and *TLR-9* in weaned piglets [44]. These findings further support the notion that bacteriophages can modulate the immune response at both the systemic and mucosal levels. Collectively, these studies provide compelling evidence that bacteriophages can modulate the immune response in weaned piglets, potentially reducing inflammation and enhancing immune function.

### 4.7. Effects on Intestinal Microecology of Weaned Piglets

One of the most well-studied applications of bacteriophages is in reducing infections caused by *E. coli* [55], a common pathogen in weaned piglets that can lead to significant morbidity and economic losses [56]. Cha et al. (2012) documented that incorporating an ETEC-specific bacteriophage into the diet led to a significant increase in bacteriophage presence in feces, as well as a notable decrease in *E. coli* loads in the feces of weaned piglets subject to ETEC JG280 challenge [34]. Similarly, Lee et al. (2017) demonstrated that dietary supplementation with an ETEC K88-selective bacteriophage decreased *E. coli* loads in the feces, jejunum, and ileum of weaned piglets challenged with ETEC K88 [30]. Consistently, Mao et al. (2023) reported that oral administration of a microencapsulated bacteriophage significantly decreased *E. coli* loads in the jejunal lymph node, cecum, and spleen, while reducing *Enterobacteriaceae* abundance and increasing *Lactobacillaceae* abundance in the duodenal microbiota of weaned piglets challenged with *E. coli* GXXW-1103 [28]. In practical production settings, bacteriophage cocktails—combinations of multiple bacteriophages targeting different strains of a pathogen—are often used to enhance efficacy and broaden the spectrum of activity [57]. Imklin et al. (2022) found that a bacteriophage cocktail administered orally reduced *E. coli* loads in the feces of weaned piglets challenged with multiple *E. coli* strains, including M170, M171, M187, M226, M242, and M243 [29]. Also, as reported by Han et al. (2016), dietary supplementation with a bacteriophage cocktail decreased ETEC K88 loads in the ileum and cecum of weaned piglets challenged with ETEC K88 and K99 [31].

*Salmonella typhimurium* is another significant pathogenic bacterium that adversely affects the growth performance and intestinal health of weaned piglets [58]. In recent years, specific bacteriophages have been explored as a targeted intervention to mitigate the negative effects of *Salmonella typhimurium* infections in weaned piglets. In a study conducted by Won et al. (2021), it was found that dietary supplementation with a *Salmonella typhimurium*-specific bacteriophage resulted in a reduction in *Salmonella typhimurium* loads in the feces and jejunum of weaned piglets challenged with *Salmonella typhimurium* CTC1110 [39]. Similarly, Thanki et al. (2022) found that dietary supplementation with a bacteriophage cocktail resulted in reduced *Salmonella* loads in the feces, stomach tissue, duodenum tissue, colon contents, and caecum contents of weaned piglets exposed to *Salmonella typhimurium* SL1344 [38]. Consistently, Seo et al. (2018) also noted that oral administration of a bacteriophage cocktail resulted in a reduction in *Salmonella* loads in the feces of weaned piglets challenged with *Salmonella typhimurium* ATCC14028 [40]. More recently, Li et al. (2024) reported that administering a bacteriophage cocktail orally resulted in a reduction in *E. coli* and *Salmonella* loads in the jejunum, as well as a decrease in *Enterobacteriaceae* abundance in the jejunal microbiome of weaned piglets exposed to *E. coli* O157:H7 and *Salmonella typhimurium* SM022 [16].

**Figure 1:**
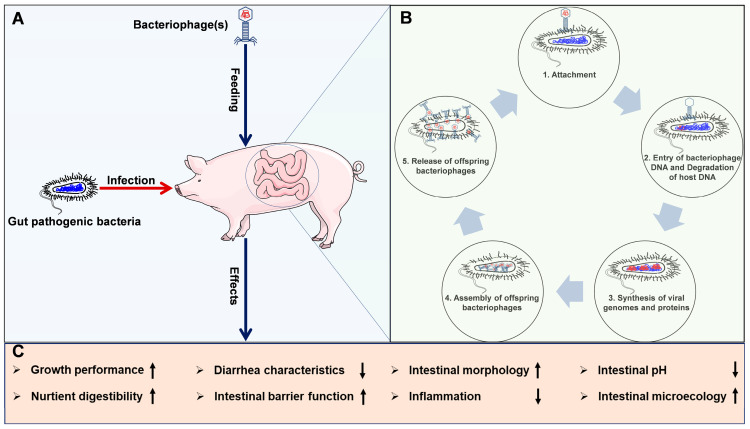
The proposed graphical summary of application of bacteriophages in weaned piglets: (**A**) the administration of bacteriophages in weaned piglets who are susceptible to intestinal pathogenic bacterial infections, such as *E. coli* and *Salmonella typhimurium*; (**B**) the lytic cycle of lytic bacteriophages; (**C**) the potential beneficial effects of bacteriophage application in weaned piglets.

Bacteriophage cocktails can selectively reduce pathogenic bacterial loads while potentially promoting the growth of beneficial microbes. In our previous study, dietary supplementation with a bacteriophage cocktail increased the α-diversity of the gut microbiota in weaned piglets, as shown by higher Observed_species, Chao1, ACE, and PD_whole_tree indices compared to piglets receiving antibiotics [44]. Bacteriophage cocktails have been demonstrated to regulate the composition of gut microbiota in weaned piglets [32,41,42,45,46,47]. Choi et al. (2023) demonstrated that a bacteriophage cocktail supplementation reduced Proteobacteria, *Megasphaera*, *Desulfovibrio*, *Schwartzia*, *Escherichia-Shigella*, *Lactobacillus_ruminis*, *Clostridium* spp., and coliforms in piglet fecal microbiota, while increasing *Eubacterium*, *Lachnospiraceae_UCG010*, *Cellulosilyticum*, and *Lactobacillus* spp. [41]. Similarly, Hosseindoust et al. (2017) noted decreased *Clostridium* spp. and coliforms in ileal microbiota and *Clostridium* spp. in caecal microbiota, with increased *Lactobacillus* spp. (ileal) and *Bifidobacterium* spp. (caecal) in weaned piglets fed a diet supplemented with a bacteriophage cocktail [42]. Liu et al. (2024) further showed that bacteriophage cocktail administration elevated *Veillonellales_Selenomonadales* and reduced *Rikenellaceae* and *Prevotellaceae_NK3B31_group* in fecal microbiota, alongside increased colonic isobutyrate and isovalerate levels in weaned piglets [32]. Additional studies have consistently highlighted the ability of bacteriophage cocktails to enhance beneficial microbial populations (e.g., *Lactobacillus* spp., *Bifidobacterium* spp.) and reduce pathogenic ones (e.g., *Clostridium* spp., coliforms) in both ileal and fecal microbiota [45,46,47]. Collectively, these studies demonstrate that bacteriophages can effectively reduce pathogenic bacterial loads, such as *E. coli* and *Salmonella typhimurium*, in weaned piglets while promoting beneficial microbial growth. This dual action enhances gut health, improves growth performance, and reduces the risk of enteric infections, highlighting bacteriophages as a promising alternative to antibiotics in weaned piglet management. The proposed graphical summary of the application of bacteriophages in weaned piglets is shown in Figure 1.

It should be noted that although bacteriophages show great promise in weaned piglets, there are still some challenges in their development and widespread application. Firstly, host specificity is the key advantage of bacteriophages in targeting specific pathogenic bacteria, which also determines their relatively narrow host spectrum [59]. Despite the proposal of bacteriophage cocktails as a partial solution to this problem [60], their application in practical pig production remains a significant challenge under complex rearing conditions. Secondly, the bacterial resistance evolution and the transmission risk of drug resistance genes are critical factors to consider for bacteriophage application in weaned piglets. Thirdly, despite the approval of bacteriophage cocktails for agricultural use by U.S. regulators in 2005, the complex and variable regulatory landscape across different regions necessitates careful consideration of regulatory issues in bacteriophage applications [61]. Lastly, the industrialization of bacteriophages represents both a prerequisite for their widespread application in piglets and a crucial future direction for this research field.

## 5. Conclusions

In conclusion, bacteriophages hold considerable promise as anti-pathogenic agents in weaned piglets. A growing body of research has demonstrated that bacteriophages exert beneficial effects on weaned piglets, particularly in enhancing growth performance, alleviating diarrhea symptoms, improving intestinal morphology, regulating intestinal pH, increasing nutrient digestibility, modulating inflammatory responses, strengthening intestinal barrier function, and regulating intestinal microecology. Given these findings, the development and application of bacteriophages represent an emerging and promising avenue for future research into diarrhea management and intestinal health in weaned piglets in the post-antibiotic era. Future endeavors should prioritize addressing key challenges, such as the narrow host range of bacteriophages, bacterial resistance evolution, and the potential transmission of antibiotic resistance genes. Furthermore, regulatory frameworks must be rigorously assessed to ensure adherence to safety and efficacy standards while advancing the industrialization of bacteriophage production to facilitate widespread application.

## Figures and Tables

**Table 1:** Isolation of specific bacteriophages targeting intestinal pathogenic bacteria in weaned piglets.

Host Bacteria Strains	Sample Sources	Screened Bacteriophages	References
1 *Escherichia coli* (*E. coli*) strain (*E. coli * GXXW-1103)	Pig farm sewage	1 bacteriophage (bacteriophage A221)	[28]
14 *E. coli* strains (*E. coli* M158, M170, M171, M179, M181, M184, M187, M209, M226, M240, M241, M242, M243, M245)	Drained water from meat stalls^1^ and dissected areas from local markets	6 bacteriophages (bacteriophage vB_EcoM-RPN170, vB_EcoM-RPN171, vB_EcoM-RPN187, vB_EcoM-RPN226, vB_EcoM-RPN242, vB_EcoP-RPN243)	[29]
1 *E. coli* strain (enterotoxigenic *E. coli* (ETEC) K88)	Pig manure from a commercial farm	1 bacteriophage (bacteriophage L86)	[30]
2 *E. coli* strains (ETEC K88 and K99)	Feces of 30- to 70-day-old grower pigs on a commercial swine farm	2 bacteriophages (ETEC K88-specific bacteriophage and K99-specific bacteriophage)	[31]
87 *E. coli* strains	Feces from a pig farm	1 bacteriophage (bacteriophage C1)	[12,32]
1 *E. coli* strain (ETEC JG280)	Pig farm sewage	1 bacteriophage (ETEC-specific lytic bacteriophage CJ12)	[33,34]
1 *E. coli* strain (*E. coli* O157:H7 GN07)	Pig farm sewage	1 bacteriophage (bacteriophage EP01)	[16,35]
1 *Salmonella typhimurium* strain (*Salmonella typhimurium* SM022)	Pig farm sewage	1 bacteriophage (*Salmonella* bacteriophage NJ12)	[16]
12 *Salmonella* strains	Food processing plant and pig farm	2 bacteriophages (bacteriophage SPFM10 and SPFM14)	[36,37,38]
1 *Salmonella typhimurium* strain	Pig manure collected from a sewage treatment plant of a commercial swine farm	1 bacteriophage (*Salmonella typhimurium*-specific bacteriophage STP-1)	[39]
6 *Salmonella* strains (*Salmonella enterica* Enteritidis SE30, *Salmonella enterica* Gallinarum SG40, *Salmonella enterica* typhimurium ST11, *Salmonella enterica* typhimurium ST2, *Salmonella enterica* Enteritidis SE5, *Salmonella enterica* Choleraesuis SC1)	Sewage and feces from a pig farm	16 lytic bacteriophages (bacteriophage SEP-1, SGP-1, STP-1, SS3eP-1, EK99P-1, SalTP-2, SChP-1, SAP-1, SAP-2, E41P-1, EK88P-1, CPP-3, CPP-5)	[40]

^1^ A meat stall refers to a specific location or booth for displaying and selling meat products.

**Table 2:** The summary of bacteriophage application in weaned piglets.

Subject	Bacteriophage	Bacteriophage Dosage and Duration	Challenge Model	Main Results (Bacteriophage Group vs. Control Group)	References
Weaned piglets (3-week-old)	Microencapsulated bacteriophage (1 bacteriophage isolated using *Escherichia coli* (*E. coli*) GXXW-1103 as host strain)	Dosage: 1 × 10^9^ plaque-forming units (PFUs)/mL, 5 mL/day (oral administration) Duration: 7 days	*E. coli* challenge (*E. coli* GXXW-1103) (the challenge group as the control group)	Growth performance: ↑ average daily feed intake (ADG); The amount of *E. coli* loads: ↓ jejunal lymph node, cecum, spleen; Duodenal microbiota: ↓ *Enterobacteriaceae*, ↑ *Lactobacillaceae*, *Oscillospiraceae*.	[28]
Weaned piglets (3-week-old)	Bacteriophage cocktail (6 bacteriophages isolated using 14 multidrug-resistant *E. coli* as host strains)	Dosage: 2 × 10^9^ and 2 × 10^10^ PFU/pig (oral administration) Duration: 3 times after *E. coli* challenge (24, 48, 72 h)	*E. coli* challenge (*E. coli* M170, M171, M187, M226, M242, M243) (the challenge group as the control group)	Growth performance: no effects; The amount of *E. coli* loads: ↓ feces.	[29]
Weaned piglets (5-week-old)	Enterotoxigenic *E. coli* (ETEC) K88-specific bacteriophage	Dosage: 1 × 10^7^ PFU/kg diet (dietary supplementation) Duration: 14 days	ETEC K88 challenge (the challenge group as the control group)	Growth performance: ↑ final body weight (BW), ADG; The amount of *E. coli* loads: ↓ feces, jejunum, ileum; Serum inflammatory factors: ↓ tumor necrosis factor-α (TNF-α); Digesta pH: ↓ colon; Intestinal morphology: ↑ ileum villous height (VH), VH/crypt depth (CD), ↓ CD; Goblet cell density: ↑ ileum villous, crypt.	[30]
Weaned piglets (4-week-old)	Bacteriophage cocktail (2 bacteriophages specific to ETEC K88 and K99, respectively)	Dosage: 1 × 10^9^ PFU each bacteriophage/kg diet (dietary supplementation) Duration: 7 days	ETEC K88 and K99 challenge (the challenge group as the control group)	Growth performance: ↑ final BW, ADG, ↓ rectal temperature, fecal consistency score; Digesta pH: ↓ colon; The amount of ETEC K88 loads: ↓ ileum, cecum.	[31]
Weaned piglets (3-week-old)	ETEC-specific bacteriophage	Dosage: 1 × 10^6^ and 1 × 10^8^ PFU/g diet (dietary supplementation) Duration: 7 days	ETEC JG280 challenge (the challenge group as the control group)	Growth performance: ↓ diarrhea percentage; The amount of *E. coli* loads: ↓ feces; The amount of bacteriophage: ↑ feces.	[34]
Weaned piglets (3-week-old)	Microencapsulated bacteriophage cocktail (EP01 and NJ12 bacteriophages isolated using *E. coli* O157:H7 and *Salmonella typhimurium* SM022 as host strains, respectively)	Dosage: 1 × 10^8^ PFU/mL each bacteriophage, 6 mL/day (oral administration) Duration: 7 days	*E. coli* O157:H7 and *Salmonella typhimurium* SM022 challenge (the challenge group as the control group)	Growth performance: ↓ diarrhea incidence; Blood parameters: ↓ neutrophil rate; The amount of *E. coli* and *Salmonella* loads: ↓ jejunum; Jejunal microbiome: ↓ *Enterobacteriaceae*.	[16]
Weaned piglets (7.28–7.49 kg)	Bacteriophage cocktail (SPFM10 and SPFM14 bacteriophages)	Dosage: 2 × 10^9^ PFU/kg, 65 kg/ton (dietary supplementation) Duration: 9 days	*Salmonella typhimurium* SL1344 challenge (the challenge group as the control group)	The amount of *Salmonella* loads: ↓ feces, stomach tissue, duodenum tissue, colon contents, caecum contents.	[38]
Weaned piglets (3-week-old)	*Salmonella typhimurium*-specific bacteriophage	Dosage: 1 × 10^9^ PFU/kg diet (dietary supplementation) Duration: 14 days	*Salmonella typhimurium* CTC1110 challenge (the challenge group as the control group)	Growth performance: ↑ final BW, ADG; The amount of *Salmonella typhimurium* loads: ↓ feces, jejunum; Jejunal morphology: ↑ VH, VH/CD; Jejunal goblet cell density: ↑ villi.	[39]
Weaned piglets (4-week-old)	Bacteriophage cocktail (SEP-1, SGP-1, STP-1, SS3eP-1, SalTP-2, SChP-1, SAP-1, and SAP-2 bacteriophages)	Dosage: 1 × 10^9^ PFU/mL, 5 mL (oral administration) Duration: 21 days	*Salmonella typhimurium* ATCC14028 challenge (the challenge group as the control group)	The amount of *Salmonella* loads: ↓ feces.	[40]
Weaned piglets (3-week-old)	Bacteriophage cocktail (*E. coli* (K88, K99, and F41), *Salmonella* (*S. typhimurium* and *S. enteritidis*), and *Clostridium perfringens* types A- and C-specific bacteriophages)	Dosage: 1 × 10^8^ PFU/kg diet (dietary supplementation) Duration: 14 days	Non-sanitary environment challenge (the challenge group as the control group)	Growth performance: ↑ ADG, gain-to-feed ratio (G/F), ↓ fecal score; Serum inflammatory factors: ↓ interleukin-1β (IL-1β), interleukin-6 (IL-6), TNF-α, Zonulin; Jejunal inflammatory enzymes: ↓ myeloperoxidase; Jejunal liver-injury enzymes: ↓ alkaline phosphatase; Intestinal morphology: ↑ duodenal VH; Fecal microbiota: ↓ Proteobacteria, *Megasphaera*, *Desulfovibrio*, *Schwartzia*, *Escherichia-Shigella*, *Lactobacillus_ruminis*, *Clostridium* spp., coliforms, ↑ *Eubacterium*, *Lachnospiraceae_UCG010*, *Cellulosilyticum*, *Lactobacillus* spp.	[41]
Weaned piglets (8.08 ± 0.36 kg)	Bacteriophage cocktail (*E. coli* (K88, K99, and F41), *Salmonella* (*S. typhimurium*, *S. enteritidis*, *S. cholerasuis,* and *S. derby*), *Staphylococcus aureus*, and *Clostridium perfringens* types A- and C-specific bacteriophages)	Dosage: 1 × 10^9^ PFU for each bacteriophage/g, 1 g/kg (dietary supplementation) Duration: 35 days	Contaminated environment challenge (the challenge group as the control group)	Growth performance: ↑ ADG, G/F, ↓ fecal score; Apparent total tract digestibility of nutrients: ↑ crude protein; Ileal microbiota: ↑ *Lactobacillus* spp., ↓ *Clostridium* spp., coliforms; Caecal microbiota: ↑ *Bifidobacterium* spp., ↓ *Clostridium* spp.; Intestinal morphology: ↑ jejunal VH.	[42]
Weaned piglets (3-week-old)	Bacteriophage cocktail (C1, S19cd, S143_2, N2, and C6 bacteriophages)	Dosage: 5 × 10^8^ PFUs of each bacteriophage per 10 mL, 10 mL/day (oral administration) Duration: 20 days	No challenge (the non-bacteriophage-administrated group as the control group)	Intestinal tight junction protein levels: ↑ zonula occludens-1 (ZO-1), Claudin-1, mucin 2 (MUC2) in the jejunum and colon; Fecal microbiota: ↑ *Veillonellales_Selenomonadales*, ↓ *Rikenellaceae*, *Prevotellaceae*_NK3B31_group, *Rikenellaceae*_RC9_gut_group; Colonic short-chain fatty acid levels: ↑ isobutyrate, isovalerate.	[32]
Weaned piglets (6-week-old)	Bacteriophage cocktail (*E. coli* (K88, F18, Stx2e [ETEC], and enteropathogenic *E. coli*), *Salmonella* (*S. typhimurium*, *S. enteritidis,* and *S. cholerasuis*), and *Clostridium perfringens* types A- and C-specific bacteriophages)	Dosage: 1 × 10^9^ PFU/g of each bacteriophage, 1 g/kg diet (dietary supplementation) Duration: 42 days	No challenge (the antibiotics group as the control group)	Growth performance: ↓ fecal score; Intestinal morphology: ↓ duodenal VH/CD, jejunal VH, CD, VH/CD.	[43]
Weaned piglets (25-day-old)	Bacteriophage cocktail (*E. coli* (K88, K99, 987P, F18, F41, and O78), *Salmonella* (*S. choleraesuis*, *S. derby*, *S. dublin*, *S. enteritidis*, *S. gallinarum*, *S. pullorum,* and *S. typhimurium*), *Clostridium perfringens* (types A, B, C, D, and E), and *Staphylococcus aureus*-specific bacteriophages)	Dosage: 1 × 10^6^ PFU/g, 0.4 g/kg (dietary supplementation) Duration: 21 days	No challenge (the antibiotics group as the control group)	Growth performance: ↑ final BW, average daily feed intake (ADFI), ADG, G/F, ↓ diarrhea incidence; Intestinal morphology: ↑ VH (jejunum, ileum), VH/CD (duodenum, jejunum, ileum), ↓ CD (jejunum, ileum); Serum parameters: ↓ IL-1β, TNF-α, D-lactate, diamine oxidase (DAO), ↑ interleukin-10 (IL-10); Ileal mucosal barrier factors: ↑ secretory immunoglobulin A (sIgA), tumor growth factor-α (TGF-α), intestinal trefoil factor (ITF); Jejunal mucosal mRNA levels: ↑ toll-like receptor-2 (*TLR-2*), toll-like receptor-4 (*TLR-4*), toll-like receptor-9 (*TLR-9*), *Occludin*, *Claudin-1*; Jejunal mucosal protein levels: ↑ Occludin; Gut microbiota: ↑ Observed_species, Chao1, ACE, PD_whole_tree.	[44]
Weaned piglets (7.27 ± 0.26 kg)	Bacteriophage cocktail (*E. coli* (K88, K99, and F41), *Salmonella* (*S. typhimurium*, *S. enteritidis*, *S. cholerasuis,* and *S. derby*), *Staphylococcus aureus*, and *Clostridium perfringens* types A- and C-specific bacteriophages)	Dosage: 1 ×10^9^ PFU/g, 1 g/kg (dietary supplementation) Duration: 35 days	No challenge (the non-bacteriophage-supplemented group as the control group)	Growth performance: ↑ ADG, G/F, ↓ fecal score; Ileal microbiota: ↑ total anaerobic bacteria, *Bifidobacterium* spp., *Lactobacillus* spp., ↓ coliforms; Caecal microbiota: ↓ *Clostridium* spp.; Fecal microbiota: ↑ *Lactobacillus* spp., ↓ *Clostridium* spp.; Apparent total tract digestibility of nutrients: ↑ dry matter, crude protein; Intestinal morphology: ↑ duodenal VH, jejunal VH.	[45]
Weaned piglets (24 ± 3-day-old)	Bacteriophage cocktail (*E. coli* (K88, K99, and F41), *Salmonella* (*S. typhimurium*, *S. enteritidis*, *S. cholerasuis,* and *S. derby*), *Staphylococcus aureus*, and *Clostridium perfringens* types A- and C-specific bacteriophages)	Dosage: 1 × 10^9^ PFU/g, 1.5 g/kg (dietary supplementation) Duration: 35 days	No challenge (the non-bacteriophage-supplemented group as the control group)	Growth performance: ↑ ADG, ↓ fecal score; Apparent total tract digestibility of nutrients: ↑ dry matter, crude protein; Ileal microbiota: ↑ *Lactobacillus* spp., ↓ coliforms, *Clostridium* spp.; Intestinal morphology: ↑ duodenal VH, jejunal VH.	[46]
Weaned piglets (7.34 ± 0.27 kg)	Bacteriophage cocktail (*E. coli* (K88, K99, and F41), *Salmonella* (*S. typhimurium*, *S. enteritidis*, *S. cholerasuis,* and *S. derby*), *Staphylococcus aureus*, and *Clostridium perfringens* types A- and C-specific bacteriophages)	Dosage: 1 ×10^9^ PFU/g, 1 g/kg (dietary supplementation) Duration: 35 days	No challenge (the non-bacteriophage-supplemented group as the control group)	Growth performance:↑ ADG, G/F, ↓ fecal score; Apparent total tract digestibility of nutrients: ↑ dry matter, crude protein; Fecal microbiota: ↑ total anaerobic bacteria, *Bifidobacterium* spp., *Lactobacillus* spp., ↓ *Clostridium* spp., coliforms; Intestinal morphology: ↑ duodenal VH, jejunal VH.	[47]

## Data Availability

Not applicable.

## Abbreviations

The following abbreviations are used in this manuscript:

ADFI	Average daily feed intake
ADG	Average daily gain
BW	Body weight
CD	Crypt depth
DAO	Diamine oxidase
*E. coli*	*Escherichia coli*
ETEC	Enterotoxigenic *E. coli*
G/F	Gain-to-feed ratio
IL-1β	Interleukin-1β
IL-6	Interleukin-6
IL-10	Interleukin-10
ITF	Intestinal trefoil factor
MUC2	Mucin 2
PFU	Plaque-forming unit
sIgA	Secretory immunoglobulin A
TGF-α	Tumor growth factor-α
TLR-2	Toll-like receptor-2
TLR-4	Toll-like receptor-4
TLR-9	Toll-like receptor-9
TNF-α	Tumor necrosis factor-α
VH	Villous height
ZO-1	Zonula occludens-1
